# Dosing Transcranial Magnetic Stimulation of the Primary Motor and Dorsolateral Prefrontal Cortices With Multi-Scale Modeling

**DOI:** 10.3389/fnins.2022.929814

**Published:** 2022-07-08

**Authors:** Zsolt Turi, Nicholas Hananeia, Sina Shirinpour, Alexander Opitz, Peter Jedlicka, Andreas Vlachos

**Affiliations:** ^1^Department of Neuroanatomy, Faculty of Medicine, Institute of Anatomy and Cell Biology, University of Freiburg, Freiburg, Germany; ^2^Faculty of Medicine, Interdisciplinary Centre for 3Rs in Animal Research, Justus-Liebig-University, Giessen, Germany; ^3^Department of Biomedical Engineering, University of Minnesota, Minneapolis, MN, United States; ^4^Center BrainLinks-BrainTools, University of Freiburg, Freiburg, Germany; ^5^Center for Basics in Neuromodulation, Faculty of Medicine, University of Freiburg, Freiburg, Germany

**Keywords:** transcranial magnetic stimulation, repetitive transcranial magnetic stimulation, electric field, multi-scale modeling, primary motor cortex, dorsolateral prefrontal cortex

## Abstract

Transcranial magnetic stimulation (TMS) can depolarize cortical neurons through the intact skin and skull. The characteristics of the induced electric field (E-field) have a major impact on specific outcomes of TMS. Using multi-scale computational modeling, we explored whether the stimulation parameters derived from the primary motor cortex (M1) induce comparable macroscopic E-field strengths and subcellular/cellular responses in the dorsolateral prefrontal cortex (DLPFC). To this aim, we calculated the TMS-induced E-field in 16 anatomically realistic head models and simulated the changes in membrane voltage and intracellular calcium levels of morphologically and biophysically realistic human pyramidal cells in the M1 and DLPFC. We found that the conventional intensity selection methods (i.e., motor threshold and fixed intensities) produce variable macroscopic E-fields. Consequently, it was challenging to produce comparable subcellular/cellular responses across cortical regions with distinct folding characteristics. Prospectively, personalized stimulation intensity selection could standardize the E-fields and the subcellular/cellular responses to repetitive TMS across cortical regions and individuals. The suggested computational approach points to the shortcomings of the conventional intensity selection methods used in clinical settings. We propose that multi-scale modeling has the potential to overcome some of these limitations and broaden our understanding of the neuronal mechanisms for TMS.

## Introduction

Transcranial magnetic stimulation (TMS), a non-invasive brain stimulation technique, induces electric fields (E-fields) in the brain that modulate the neuronal activity (Barker et al., 1985; Thielscher et al., 2015). The intracranial E-field properties (i.e., strength and direction) are important determinants of the biological responses to TMS (Fox et al., 2004; Opitz et al., 2013; Bungert et al., 2017; Aonuma et al., 2018; Laakso et al., 2018; Weise et al., 2020; Numssen et al., 2021). Repetitive TMS (rTMS) produces periodic E-fields and can induce lasting aftereffects in brain activity and function (e.g., cortical excitability and cognition) (Pascual-Leone et al., 1991; Huang et al., 2005; Fitzgerald et al., 2006; Hamada et al., 2007). Linking the stimulation parameters to the induced physiological effects is a crucial yet challenging step toward a better understanding of the neural mechanisms of TMS and rTMS-based therapies (Krieg et al., 2015; Sahlsten et al., 2015; Lenz et al., 2016; Beynel et al., 2020; Zmeykina et al., 2020; Turi et al., 2021b).

The vast majority of rTMS studies define and standardize stimulation intensity using the motor threshold (MT) or fixed (FXD) intensity selection approaches (Turi et al., 2021a). In the MT approach, the stimulation intensity is typically determined in a hand muscle (e.g., the first dorsal interosseous muscle) through the application of a set of single-pulse stimuli over the corresponding region of the primary motor cortex (M1). The stimulation intensity is expressed as the maximum stimulator output percentage (MSO%) required to produce a given number of motor-evoked potentials (MEPs) with a minimum amplitude *via* the pulse sequence (e.g., 5 MEPs out of 10 TMS pulses) (Borckardt et al., 2006).

Because MT intensities vary among participants, this approach individually adjusts the stimulation intensity to induce MEPs. Hence, the standardized rTMS treatments stimulate the target region (typically not the M1) at a given percentage of the MT intensity. For example, the US Food and Drug Administration (FDA)-approved rTMS protocols for the treatment of pharmacoresistant depression use a 120% MT intensity to stimulate the dorsolateral prefrontal cortex (DLPFC) (George et al., 2010; Blumberger et al., 2018). By contrast, the FXD approach relies on the “one size fits all” concept and uses a non-individualized, fixed intensity (e.g., 55 or 60% MSO) (Schaal et al., 2015; Ambrus et al., 2020).

Studies using rTMS frequently target non-motor cortical regions while deriving some stimulation parameters (e.g., intensity and location) from the M1 (e.g., Matsuura et al., 2015; Strang et al., 2015; Xie et al., 2020). However, the properties of the cortical regions can differ in several aspects. These differences include the scalp-to-cortex distance as well as the cortical folding pattern and neuronal orientation, among others (Stokes et al., 2005; Lützkendorf et al., 2018; Schmitz et al., 2019; Castrillon et al., 2020). In addition, the MSO% reported in the literature for the MT approach frequently exhibits substantial individual variability (Laviolette et al., 2013; Matsuura et al., 2015; Strang et al., 2015; Xie et al., 2020; Zmeykina et al., 2020). Differences in these factors may affect the induced macroscopic E-field properties (Janssen et al., 2015; Gomez-Tames et al., 2018) and modulate the functional effects of rTMS. Therefore, producing comparable biological effects across cortical regions using the same set of stimulation parameters may be difficult to achieve.

**Figure 1:**
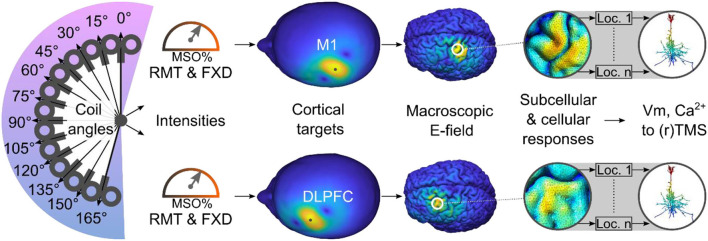
Study overview. Using a multi-scale computational approach, we modeled the TMS-induced macroscopic E-field and the subcellular/cellular responses to (r)TMS in the M1 and DLPFC using conventional stimulation intensities. Ca^2+^, calcium concentration; DLPFC, dorsolateral prefrontal cortex; FXD, fixed stimulation intensity; Loc., location of cell; M1, primary motor cortex; MSO%, maximum stimulator output percentage; RMT, resting motor threshold; Vm, membrane voltage.

Recently, multi-scale computational frameworks have been developed to model the subcellular and cellular responses of single neurons to realistic E-fields produced by TMS (e.g., Seo et al., 2016; Seo and Jun, 2019; Aberra et al., 2020; Shirinpour et al., 2021) and rTMS (Shirinpour et al., 2021). The current study builds on these recent advances by employing multi-scale computational modeling to investigate the physiological effects in two commonly selected cortical regions. We selected the M1 as one of the cortical targets because most studies adjust the stimulation intensity according to the MT intensity (Turi et al., 2021a). The DLPFC was chosen as the other cortical region because it is commonly targeted in a wide range of rTMS applications, including cognitive and affective research (e.g., Beynel et al., 2019) and clinical treatments [e.g., for depression (Iimori et al., 2019)]. Using this multi-scale computational modeling approach, we assessed whether the M1-derived stimulation parameters would induce macroscopic E-field strengths and subcellular/cellular responses in the DLPFC comparable to those elicited by the conventional intensity selection approaches (i.e., MT and FXD; Figure 1).

## Materials and Methods

### Study Overview

In total, 16 anatomically realistic head models were generated from an openly available MRI dataset, which also includes resting motor threshold (RMT) data for each individual (https://github.com/ZsoltTuri/2019_rTMS-EEG) (Zmeykina et al., 2020). This unique database enables the modeling of the E-field produced by the conventional RMT-based intensity selection approach as well as other conventional (i.e., the FXD-approach) and non-conventional (i.e., prospective E-field modeling) approaches in both cortical targets (i.e., M1 and DLPFC), respectively (Turi et al., 2021a).

### Research Integrity

The code for performing the modeling and analyses for the present study are available for download at the repository (https://github.com/ZsoltTuri/2021_human_multi_scale_modeling).

### Macroscopic E-Field Simulations

We used the open-access toolbox called Simulation of Non-invasive Brain Stimulation (SimNIBS; version 3.1.2) to create head models and perform all E-field simulations (Thielscher et al., 2015). Simulations were run on a Windows machine (Windows 10 Pro, version 1909). We called the “headreco()” SimNIBS function with “–no-cat” flag to create anatomically realistic, multi-compartmental (e.g., gray matter, white matter, etc.) head models (Nielsen et al., 2018). To improve the smoothness of the skin compartment, we increased the number of cortical smoothing repetitions from the default value of five to 200. In all cases, we manually approved the segmentation and corrected the segmentation inaccuracies in the skin and bone compartments using the free program package called ITK-Snap (version 3.8.0) (Yushkevich et al., 2006). After manual segmentation, we called “surfacemesh()” and “volumemesh()” SimNIBS functions to create the final head meshes. For each compartment, we used the default conductivity values (in S/m): eyes (0.5), scalp (0.465), bone (0.01), cerebrospinal fluid (1.654), gray matter (0.275), white matter (0.126).

We defined the anatomical target in the M1 based on a previous meta-analysis (Mayka et al., 2006). First, we converted the M1 from the Montreal Neurological Institute coordinate space (MNI152; *x* – 37, *y* – 21, *z* + 58) into subject space. Then, we computed the Euclidean distance between the M1 coordinate in the gray matter compartment and the scalp compartment and selected the scalp position corresponding to the shortest distance to the stimulation target in the precentral gyrus. The stimulation target in the DLPFC was defined relative to the M1 using the “5-cm rule” (i.e., the coil was moved 5 cm anterior to the M1 target location) which is a common clinical method (George et al., 2010). Similar to the M1 target, we chose the shortest Euclidean distance between the scalp and gray matter compartments to define the ROI center at the DLPFC.

Note that the individual coordinates for the first dorsal interosseous muscle hot spots are not available in the database. Table 1 provides an overview of RMTs and cortical depth values (i.e., the distance between the coil center and gray matter ROI center) extracted from the SimNIBS output log file.

**Table 1:** Overview of participant data.

		**Cortical depth (mm)**	
**ID**	**RMT (MSO%)**	**M1**	**DLPFC**
1	75	15.88	16.89
2	68	15.76	15.45
6	75	14.03	13.74
8	64	12.30	12.10
9	46	15.07	12.88
12	42	10.89	12.30
13	75	18.44	17.52
14	47	13.60	12.83
15	69	16.12	14.27
16	52	13.96	12.41
17	72	13.52	14.65
19	43	12.05	10.13
21	41	14.12	12.65
22	56	18.33	21.46
24	47	11.89	10.78
25	75	16.48	13.85

*Cortical depths correspond to the distance in mm between the coil center and the ROI center in the gray matter compartment. RMT values are expressed as MSO%. IDs were taken from the original database*.

*DLPFC, dorsolateral prefrontal cortex; M1, primary motor cortex; MSO%, maximum stimulator output percentage; RMT, resting motor threshold*.

For all simulations, we set the stimulation intensity at a coil-current rate of change of 1.49 A/μs that corresponded to 1% MSO (MagPro X100 stimulator and MC-B70 coil, MagVenture, Denmark) and used a four mm scalp-to-coil distance. The normal current direction in this device generates a posterior-to-anterior E-field direction in the brain for the initial phase and an anterior-to-posterior E-field direction in the second phase of the TMS pulse when using a ca. 45–60° coil rotation angle with respect to longitudinal fissure and the coil handle is pointing posteriorly. To study the inter-individual variability produced by the conventional intensity selection approaches, we scaled the extracted E-field values, originally estimated at 1% MSO, the intensity with the corresponding stimulation intensities (based on individual RMT or fixed MSO values). This was possible because the magnitude of the induced E-field is proportional to the coil-current rate of change value, and we only manipulated the stimulation intensity parameter while keeping all other stimulation parameters (i.e., coil-to-cortex distance, coil center position, and rotation angle) constant. At each cortical target, we systematically manipulated the coil's rotation angle between 0° and 165° in 15° steps.

We extracted the mean E-field strength using a circular region of interest (ROIs) with a 10 mm radius. The ROI center was located at the closest point in the gray matter surface relative to the coil's center position. In addition to the mean E-field, we also extracted the robust maximum (i.e., 99.9th percentage) of the produced E_TOTAL_.

### Multi-Scale Computational Modeling

We used the Neuron Modeling for TMS (NeMo-TMS) framework to study the cellular response after single-pulse TMS and rTMS (Shirinpour et al., 2021). Due to the high computational demand, we ran all simulations on a high performance computer in the state of Baden-Württemberg, Germany (bwHPC). We used the following toolboxes for the simulations (version): NEURON (8.0.0), TREES (1.15), T2N (1.9), VRL (0.4.4.0.3), VRL studio (0.4.8.0), MATLAB (2020), Gmsh (3.0.6), and SimNIBS (3.2.4).

All simulations were performed using a layer five human neocortical neuron and biphasic TMS pulses (Shirinpour et al., 2021). Both in M1 and DLPFC, the cells were oriented perpendicular to the cortical surface with a somatic depth of 1.25 mm. The rationale for choosing this somatic depth was to ensure that all neuronal compartments remained within the gray matter compartment (e.g., dendrites were not penetrating into the CSF compartment). We set the ROI radius to be 15 mm and populated neurons in this region (mean ± SD in DLPFC = 1,279 ± 177.10; M1 = 1,419 ± 149.25). Here, we used a slightly larger radius for the ROI than for the macroscopic E-field modeling, to study whether single-pulse TMS could activate neurons slightly further away from the coil.

To avoid the high computational cost, due to a large number of simulation parameters [2 cortical targets × 12 coil orientations × 1,348.60 ± 176.15 (mean ± standard deviation) cell locations × 16 participants], we limited the analysis to the 45° coil angle positions for each cortical target. In all analyses, we extracted the membrane potentials from the somatic compartment. We estimated the activation threshold at each neuronal location as the minimum stimulation intensity needed to generate time-locked action potentials to a single TMS pulse at 1% MSO precision.

We added a synapse to the pyramidal cell 20 μm away from the soma on the apical dendrite. The two-state kinetic scheme synapse can be described by rise time τ1 (0.2 ms), and decay time constant τ2 (2.5 ms). We estimated the synaptic threshold as the minimum synaptic weight needed to generate action potentials in the cell without the application of TMS. This value corresponds to 0.13 μS. We ran simulations with and without synaptic inputs. For the simulations with weak synaptic inputs, we used 10% of the synaptic threshold. The synaptic input slightly reduced the activation threshold of the given neuron without inducing action potentials on its own. As expected, the activation threshold was lower for neurons with synaptic inputs. For single-pulse TMS, the remaining statistical analyses focused on the series of simulations that involved neurons with weak synaptic input.

### Statistical Analysis

We analyzed the data using R (version 4.0.3) (R Core Team, 2020) and the R studio integrated development environment (version 1.3.1093) (RStudio Team, 2020). We ran the Shapiro–Wilk's normality test to see whether the dependent variable showed any significant deviation from normality. For normally distributed data, we used two-sided paired samples *t*-test for pairwise comparisons. Otherwise, we ran the Wilcoxon two-sample paired signed-rank test. We reported all *p*-values after Bonferroni–Holm adjustment.

We used linear mixed-effect models implemented in the lmerTest R library (Kuznetsova et al., 2017). For the macroscopic E-field data, we studied the effects of the cortical target (two levels: M1, DLPFC), coil angle (12 levels: 0–165° with 15° steps), and their interaction on the E-field strength. To study the effect of the cortical target (two levels: M1, DLPFC) on the activation threshold (expressed in MSO%) of the cells, we ran generalized mixed-effect models (the Poisson regression with logit link function). For all models, we set the factor participant as a random intercept. To study the effect of the cortical target on the activated neurons, we ran a binomial mixed-effect model.

We used the Bayesian information criterion (BIC) for model selection. We selected the winning model if BIC was 6–10 lower than the null or the less complex model. We reported partial Eta squared ($\eta_{p}^{2}$) effect size values for the generalized linear mixed-effect models and paired samples *t*-test using the “effectsize” R library (Ben-Shachar et al., 2020). For the Wilcoxon two-sample paired signed-rank test, we calculated the Wilcoxon effectsize “*r*” using the “rstatix” R library (Kassambara, 2021).

## Results

### Macroscopic E-Field Simulations

**Figure 2:**
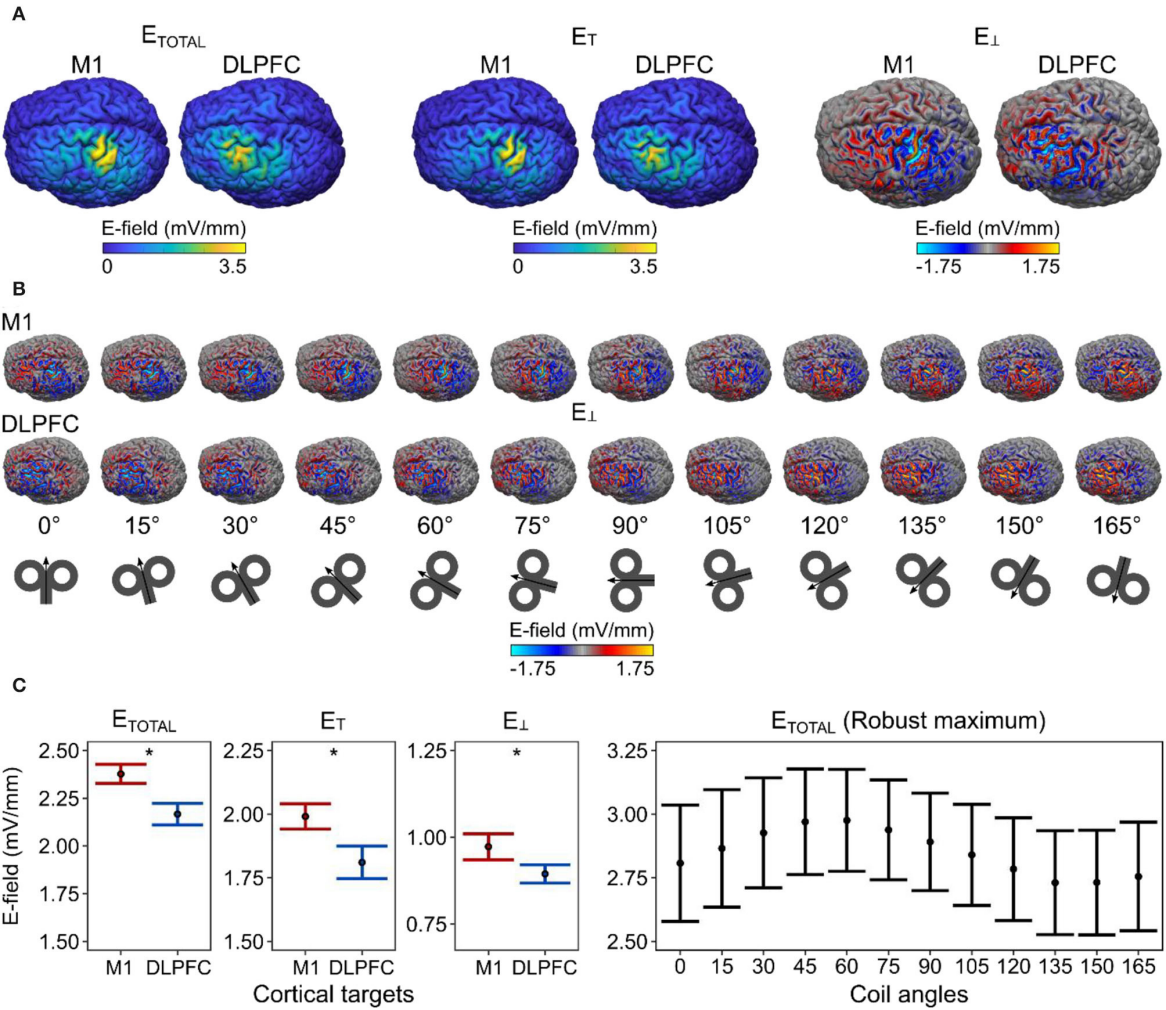
Differences in macroscopic E-field strengths between M1 and DLPFC. **(A)** The spatial distribution of the E-field values and **(B)** the effect of coil angle on the E-field distribution projected over the gray matter surface in one example participant (ID = P21). **(C)** We observed a significantly stronger mean E-field in the M1, compared with the DLPFC. The robust maximum of the E_TOTAL_. E-field values correspond to gray matter surface compartment. Dots represent the mean, error bars correspond to 95% CIs. E-field values correspond to simulations run at 1% MSO. Asterisks indicate a significant effect of factor cortical target.

We analyzed 384 simulations for the macroscopic E-field modeling (two cortical targets × 12 coil angles 16 participants), focusing on the mean total E-field strength (E_TOTAL;_ corresponding to the length of E-field vector) and it's tangential (E_T_) and perpendicular (E_⊥_; i.e., normal or radial) components with respect to the cortical surface (Figure 2A). For demonstration purposes, we showed the spatial distribution of E_⊥_ over the cortical surface at each coil angle (Figure 2B).

We ran linear mixed-effect models to study the effects of the cortical target, the coil angle, and their interaction on the produced mean E-field strength. For the E_TOTAL_, the winning model [ΔBIC = 106.190; *x*^2^(1) = 112.140, *p* = 2.00 × 10^−16^] included only the cortical target as a predictor; this model revealed that the cortical target had a significant effect on the E_TOTAL_ [*F*_1, 367_ = 130.750, *p* < 2.00 × 10^−16^, $\eta_{p}^{2}$ = 0.260, 90% CI = (0.20, 0.32)]. This result indicates that the E_TOTAL_ was significantly stronger in the M1 than in the DLPFC (Figure 2C). Likewise for E_T_, and E_⊥_, the best models [E_T_, ΔBIC = 58.942; *x*^2^(1) = 64.892, *p* = 7.91 × 10^−16^; E_⊥_, ΔBIC = 6.643; *x*^2^(1) = 12.594, *p* = 0.00039] revealed that the cortical target had a significant effect on the respective parameters [E_T_, F_1, 367_ = 70.772, *p* < 9.04 × 10^−16^, $n_{p}^{2}$ = 0.16, 90% CI = (0.11, 0.22); E_⊥_, F_1, 367_ = 12.778, *p* = 0.00040, $n_{p}^{2}$ = 0.030, 90% CI = (0.01, 0.07)].

Because previous studies mainly focused on the maximum rather than the mean E-field values in the M1, we also analyzed the robust maximum of E_TOTAL_ as a function of the coil angle. We found that a coil angle of ~45° produced the strongest E_TOTAL_ in the M1 cortical surface and volume compartments (Figure 2C, right). This is in line with previous empirical observations (e.g., Mills et al., 1992) although a modeling study has suggested that the shape of the M1 may further influence the most optimal coil angle on an individual level (Opitz et al., 2013).

We also compared the scalp-to-cortex distance between the M1 and DLPFC using a paired-samples *t*-test, which revealed no significant difference between the two cortical targets in our sample [*t*_15_ = 1.383, *p* = 0.18690, $n_{p}^{2}$ = 0.910, 90% CI = (0.00, 0.99)]. This indicates that the observed differences in the mean E-field strengths were not due to differences in the scalp-to-cortex distance in our sample (Stokes et al., 2005).

**Figure 3:**
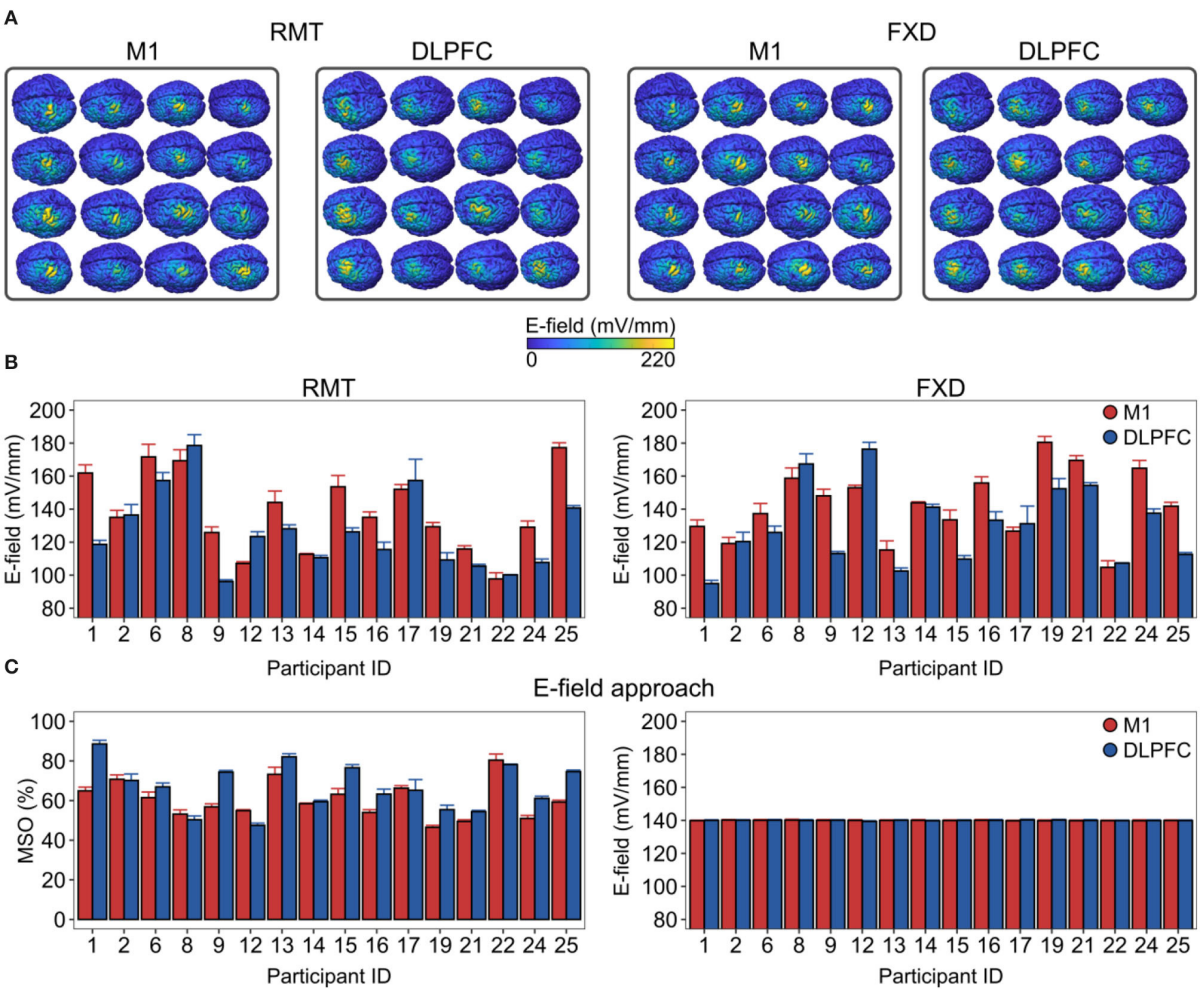
Inter-individual variability in the macroscopic E-field is produced by the conventional intensity selection approaches and its reduction by a personalized intensity adjustment method. **(A)** The spatial distribution of E_TOTAL_ over the cortical targets is shown for each individual. **(B)** There was substantial E-field variability in both conventional intensities. **(C)** We adjusted the stimulation intensities (left side) to produce closely matched macroscopic E-fields (right side) between cortical targets and across participants in the ROI. E-field values correspond to gray matter surface compartments. DLPFC, dorsolateral prefrontal cortex; FXD, fixed stimulation intensity; M1, primary motor cortex; MSO%, maximum stimulator output percentage; RMT, resting motor threshold.

Next, we estimated the optimal coil angle (i.e., the angle that produced the strongest mean E-field strength) for a given cortical target and individual and compared the resulting E-field strengths for the two cortical targets. We observed significantly stronger E_TOTAL_ values in the M1 compared with those in the DLPFC [t_15_ = 2.562, *p* = 0.04332, Bonferroni-Holm adjusted *p*-value, $n_{p}^{2}$ = 0.300, 90% CI = (0.03, 0.56); Figure 3. For the E_T_, we observed that the E-field over the M1 was stronger, although not significantly, than that over the DLPFC [*t*_15_ = 1.868, *p* = 0.08138, the Bonferroni–Holm adjusted *p*-value, $n_{p}^{2}$ = 0.190, 90% CI = (0.00, 0.46)]. For E_⊥_, we observed that the E-field over the M1 was significantly stronger than that over the DLPFC [*t*_15_ = 3.259, *p* = 0.01586, the Bonferroni–Holm adjusted *p*-value, $n_{p}^{2}$ = 0.410, 90% CI = (0.10, 0.64)].

We then focused on the inter-individual variability in the mean E-field strengths produced by conventional intensity selection approaches (Figure 3). Here, we scaled the resulting E-field according to the individual RMT intensities or using the FXD approach at 60% MSO. We found that both approaches produced substantial variability in the E-field strength (Figure 3A). For the RMT approach, the produced E_TOTAL_ ranged from 97.7 to 177 ^mV^/_mm_ in the M1 and from 96.1 to 179 ^mV^/_mm_ in the DLPFC. For the FXD approach, the E_TOTAL_ ranged from 105 ^mV^/_mm_ to 180 ^mV^/_mm_ in the M1 and from 94.9 to 176 ^mV^/_mm_ in the DLPFC (Figure 3B).

Finally, we assessed the extent to which the mean E_TOTAL_ strength could be standardized between cortical targets and among participants. To this aim, we linearly scaled the stimulation intensity (coil current rate of change) in each participant until the target E_TOTAL_ was reached in the ROI (Figure 3C, left). For illustration purposes, we set the target E_TOTAL_ conveniently at 140 ^mV^/_mm_. This approach was able to significantly reduce the inter-individual variability and the variability between cortical targets (M1 mean ± SD = 139.94 ± 0.66 ^mV^/_mm_, from 138.55 to 141.41 ^mV^/_mm_; DLPFC mean ± SD = 139.98 ± 0.63 ^mV^/_mm_, from 138.59 to 141.32 ^mV^/_mm_).

### Multi-Scale Computational Modeling

**Figure 4:**
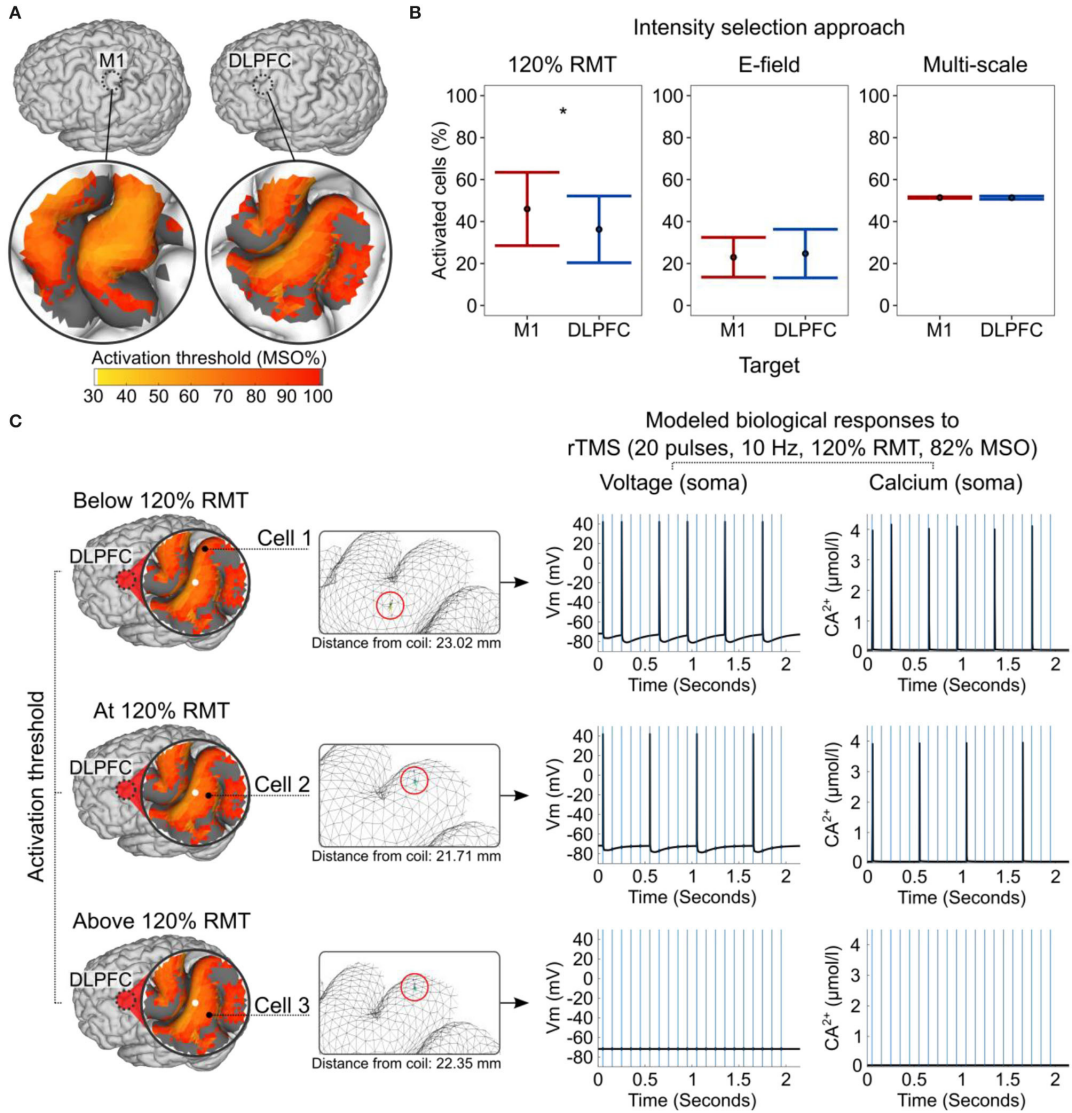
Modeled subcellular/cellular responses to single-pulse TMS and rTMS. **(A)** The activation threshold is expressed in MSO% of cells produced by single-pulse TMS and shown in an example participant (ID = 2). Dark gray color corresponds to an activation threshold above 100% MSO. **(B)** Percentage of activated cells produced by the different intensity selection approaches at the group level. **(C)** Concurrent biological responses to 10 Hz rTMS at 120% of RMT (82% MSO) in the DLPFC, depending on the cells' activation thresholds and locations within the cortical folding shown in an example participant (ID = 2). A white circle indicates the coil center projected on gray matter surface. The mesh insets (in the middle) are rotated for better cell visualization. Red circles in mesh insets highlight cell locations. Blue vertical lines (right) indicate the timing of rTMS pulses and the black line shows the modeled neuronal response. DLPFC, dorsolateral prefrontal cortex; M1, primary motor cortex; MSO%, maximum stimulator output percent; RMT, resting motor threshold. Asterisks indicate a significant effect of factor cortical target.

We modeled the physiological response to TMS and rTMS using a morphologically realistic human pyramidal cell with adjusted biophysical properties (Shirinpour et al., 2021). First, we estimated the stimulation intensity needed to induce time-locked action potentials to single-pulse TMS for distinct neuronal locations in a given target region (see Figure 4A). This constellation of parameters resulted in 43,156 neuronal locations [2 cortical targets × 1,348.60 ± 176.15 (mean ± SD) neuronal locations × 16 participants]. Moreover, we simulated neuronal responses with and without weak synaptic input. Hence, we ran 86,312 simulations (i.e., 2 × 43,156) in total.

We used a generalized mixed-effect model to study the effects of the cortical target on the activation threshold. In line with the macroscopic E-field modeling results, the action potential threshold was significantly lower in the M1 than in the DLPFC (*Z* = −48.77, *p* < 2 × 10^−16^; Figure 4A). Likewise, single-pulse TMS at a given MSO% activated more neurons in the M1 than in the DLPFC (*Z* = −34.947, *p* < 2 × 10^−16^).

Next, we modeled the effects of the three intensity selection approaches and estimated the percentages of activated cells these approaches produced in each cortical target and individual. In the remaining part of the analysis, we focused on the cells with activation thresholds at or below 100% MSO. In the RMT approach, the single-pulse TMS at 120% RMT intensity activated significantly more cells in the M1 than in the DLPFC (*W* = 133, *p* = 0.00046, *n* = 16, *r* = 0.286, the Bonferroni–Holms adjusted *p*-value; Figure 4B). In addition, applying ~110% RMT to the DLPFC activated the same mean percentage of cells as 100% RMT did in the M1. This finding indirectly supports the rationale behind the common convention of using higher stimulation intensities (typically 120% RMT) for the DLPFC (Blumberger et al., 2018). The difference between the two cortical targets became non-significant when using E-field-adjusted intensities (*W* = 62, *p* = 0.80740, *n* = 16, the Bonferroni–Holms adjusted *p*-value; see also macroscopic E-field simulations). We then assessed whether using stimulation intensities corresponding to the individually estimated median activation thresholds of neurons could standardize the physiological responses. Indeed, this approach closely matched the percentages of activated neurons in the two cortical targets and produced optimal results (*W* = 85, *p* = 0.80740, *n* = 16, the Bonferroni–Holms adjusted *p*-value; Figure 4B).

We studied the neuronal effects of rTMS (i.e., membrane voltage and Ca^2+^ concentration level) in the somatic compartment. Here, we focused on modeling three scenarios, namely, when the cell's activation threshold was slightly below (i.e., 5%), exactly at, or slightly above (i.e., 5%) the 120% RMT stimulation intensity (Figure 4C). The rTMS protocol consisted of 20 TMS pulses at a 10 Hz frequency and 120% RMT intensity. Due to the enormous computational demand, we performed these simulations using a single head model and neurons without synaptic inputs. The results of these simulations revealed that—depending on the E-field properties, the convolutional characteristics of the cortical folding, and the activation threshold of the neuron—a given stimulation protocol is likely to produce various biological effects simultaneously, even close to the coil. In the studied ROI, neurons in the “optimal” locations produced more action potentials and Ca^2+^ spikes (e.g., cells 1 and 2) than neurons in “suboptimal” locations only a few millimeters apart (e.g., cell 3; Figure 4C). Due to neuronal hyperpolarization, we did not observe action potentials after every TMS pulse even at the optimal location.

## Discussion

In the present study, we used a multi-scale computation modeling approach to study the macroscopic E-field properties and the subcellular/cellular responses to TMS in two frequently chosen cortical regions using conventional intensity selection approaches. Regarding the macroscopic E-field modeling, we found that otherwise, identical stimulation parameters produced significantly stronger mean E-fields in the M1 than in the DLPFC, conceptually confirming previous findings (e.g., Gomez-Tames et al., 2018). On an individual level, the macroscopic E-field strength varied substantially when using conventional intensity selection approaches. A personalized intensity adjustment method based on prospective multi-scale modeling (based on the individually estimated median activation thresholds of neurons) could substantially reduce this inter-individual variability.

Increasingly, studies suggest that rTMS produces variable E-field strengths in the human cortex. Some of these studies have argued that standardization of the E-field strength between two cortical regions may reduce the response variability to rTMS (Zmeykina et al., 2020; Caulfield et al., 2021; Turi et al., 2021b). However, even when the mean E-field strengths between two cortical targets are closely matched, differences in the physiological responses to rTMS may arise because of differences in the spatial E-field distribution. Focusing only on the mean (or maximum, etc.) of the E-field distribution may obscure subtle but biologically relevant effects that arise from the intricate relationships between the local E-field strength, its direction, and the neuronal location and orientation within the cortical folding. Macroscopic E-field modeling alone cannot appropriately address these relationships. For this reason, we used multi-scale computational modeling to better understand the cellular and subcellular effects of TMS and rTMS.

The key findings of the multi-scale modeling were that TMS did not activate neurons homogenously in the cortical folding, even at cellular locations close to the coil (Figure 4A). Instead, in most cases, we observed maximal neuronal activation in the gyral rim and crown regions, where the E_TOTAL_ was the highest. The present results are conceptually compatible with the findings of Numssen et al. (2021), who found that the peak-to-peak amplitudes of MEPs can be best explained by the E_TOTAL_ and E_T_ strength in the gyral rim and crown regions in the precentral gyrus. This location corresponded to the premotor cortex in 13 out of 14 participants in their sample. However, our simulations indicated that our findings cannot be trivially explained by the observation that E-field intensities in deeper cortical regions (i.e., the gyral wall and sulcal fundus) are weaker than those in superficial cortical regions (i.e., the gyral crown). Our simulations demonstrated that a given cell could have a lower activation threshold even when it is located a few millimeters further away from the coil (see cell 1 vs. cell 3 in Figure 4C). These findings reveal that in addition to the intensity, both the E-field direction and the orientation of neurons within the E-field must be considered because they can have a major impact on the induced biological effects.

Another key finding of the present study was that the conventional intensity selection approaches activated different numbers of cells in the cortical targets. Specifically, 120% RMT activated a significantly higher number of cells in the M1 than in the DLPFC, and this intensity was characterized by substantial inter-individual variability (Figure 4B, left). Conversely, we could effectively standardize the number of activated cells across participants and cortical targets using alternative dosing approaches that relied on E-field (i.e., using standardized field strengths) or multi-scale modeling (i.e., using median cell activation threshold intensities; Figure 4B, right).

Currently, the FDA-approved rTMS protocols for the treatment of pharmacoresistant depression derive some of their key stimulation parameters (e.g., intensity and target location) from the M1 (George et al., 2010; Blumberger et al., 2018). Although increasing the stimulation intensity from 100 to 120% RMT seems to be a reasonable choice for the DLPFC (also confirmed by our modeling results), the functional and therapeutic relevance of using M1-derived parameters in non-motor cortical regions is still not completely understood. At the same time, using the standard correction methods (e.g., scalp-to-cortex distance correction together with the 120% RMT intensity selection) may be a reasonable choice when E-field simulations are not available. Our results suggest that part of the variability in response to rTMS therapy may stem from the parameter selection methods in current use; the conventional intensity selection approaches produced substantial variability in the modeled macroscopic E-field and subcellular/cellular responses to TMS and rTMS. This pattern is expected to influence the expression of synaptic plasticity and may thus affect the therapeutic response to rTMS. We propose that a simulation-based standardization of E-field strengths and predicted biological effects in individual patients could improve the optimization of rTMS therapies [e.g., for depression (George et al., 2010; Blumberger et al., 2018)].

Future studies are needed to test whether standardizing the average E-field intensity, its spatial distribution, or the fraction of activated cells is sufficient to improve rTMS outcomes. Regarding the simulated biological effects, we concede that current computer models do not consider distinct neuronal morphologies, neuron types (e.g., interneurons), neural connectivity and circuits, or plasticity rules. Given that the rTMS-induced biological effects emerge as a network phenomenon that ideally outlasts the stimulation duration, these aspects must be addressed in the future. Additional limitations are that the present database contains only 16 participants, and in four of these participants, the RMT was above 75% MSO. Therefore, RMT could not be determined, as it was necessary to avoid inducing perceptual discomfort due to cranial muscle activation. Nevertheless, we included data from these participants because it is common to set a maximum stimulation intensity threshold for rTMS at a given MSO% for safety reasons (e.g., Smittenaar et al., 2013).

Furthermore, it has yet to be determined whether the diseased human brain responds to rTMS in the same manner as the healthy brain. For example, the threshold for inducing synaptic plasticity may change under pathological conditions (e.g., because of changes in the excitation/inhibition balance). Our simulations suggest that slightly increasing the synaptic input can reduce the stimulation intensity needed by active neurons in a given location. Currently, we lack a comprehensive understanding of the role of distinct forms of plasticity under pathological conditions and the impact of rTMS on these forms of plasticity (Lenz and Vlachos, 2016; Galanis and Vlachos, 2020).

The implementation of additional cell types (including glial cells), neural circuits, and plasticity rules in the current computational framework provides an interesting future path toward improved model predictions that can be tested in animal models and humans (Ebner et al., 2019). These improved models will contribute to our mechanistic understanding of rTMS-based interventions and may help devise novel stimulation parameter selection approaches. The results of the present study indicate that prospectively personalized multi-scale modeling is a suitable tool for standardizing the E-field properties to achieve comparable (simulated) biological responses across cortical regions and individuals.

## Data Availability Statement

The datasets presented in this study can be found in online repositories. The names of the repository/repositories and accession number(s) can be found below: https://github.com/ZsoltTuri/2021_human_multi_scale_modeling.

## Ethics Statement

The original study was reviewed and approved by the Ethics Committee of the University Medical Center Göttingen (Application number: 35/7/17) (Zmeykina et al., 2020). Written informed consent was obtained from all participants for their participation in this study.

## Author Contributions

Conceptualization, funding acquisition, and project administration: ZT and AV. Formal analysis, investigation, methodology, and visualization: ZT. Software: ZT, NH, SS, and PJ. Supervision: AO, PJ, and AV. Writing—original draft: ZT and AV with the contribution of all the authors. All authors contributed to the article and approved the submitted version.

## Funding

This study was supported by the NIH Grants (1R01NS109498-01A1 to AO and AV; RF1MH117428 to AO), BMBF Grant (01GQ1804B to PJ), and by the Faculty of Medicine of the University of Freiburg (TUR217/21 to ZT).

## Conflict of Interest

The authors declare that the research was conducted in the absence of any commercial or financial relationships that could be construed as a potential conflict of interest.

## Publisher's Note

All claims expressed in this article are solely those of the authors and do not necessarily represent those of their affiliated organizations, or those of the publisher, the editors and the reviewers. Any product that may be evaluated in this article, or claim that may be made by its manufacturer, is not guaranteed or endorsed by the publisher.
